# Pre-Surgery Patient Health Contributes to Aggravated Sino-Nasal Outcome and Quality of Life after Pituitary Adenomectomy

**DOI:** 10.3390/medicina59010127

**Published:** 2023-01-09

**Authors:** Witold X. Chmielewski, Sebastian Walbrodt, Laurèl Rauschenbach, Mehdi Chihi, Oliver Gembruch, Marvin Darkwah Oppong, Sebastian Schroer, Karsten H. Wrede, Philipp Dammann, Ramazan Jabbarli, Ilonka Kreitschmann-Andermahr, Taku Sato, Nicole Unger, Stefan Mattheis, Ulrich Sure, Yahya Ahmadipour

**Affiliations:** 1 Department of Neurosurgery and Spine Surgery, University Hospital Essen, 45147 Essen, Germany; 2 German Cancer Consortium (DKTK) Partner Site, University Hospital Essen, 45147 Essen, Germany; 3 Center for Translational Neuro- & Behavioral Sciences (C-TNBS), University Duisburg Essen, 47147 Duisburg, Germany; 4 Department of Neurosurgery, Fukushima Medical University, Fukushima 960-1247, Japan; 5 Department of Endocrinology, Diabetes and Metabolism, University Hospital Essen, 45147 Essen, Germany; 6 Department of Otorhinolaryngology, Head and Neck Surgery, University Hospital Essen, 45147 Essen, Germany

**Keywords:** pituitary adenoma, adenomectomy, sino-nasal health, health-related quality of life, pre-surgery patient health

## Abstract

*Objectives*: The transphenoidal bi-nostril endoscopic resection of pituitary adenomas is regarded as a minimally invasive treatment nowadays. However, sino-nasal outcome and health-related quality of life (HRQoL) might still be impaired after the adenomectomy, depending on patients’ prior medical history and health status. A systematic postoperative comparison is required to assess differences in perceived sino-nasal outcome and HRQoL. *Methods*: In this single-center observational study, we collected data from 81 patients, operated between August 2016 and August 2021, at a 3–6-month follow-up after adenomectomy. We employed the sino-nasal outcome test for neurosurgery (SNOT-NC) and the HRQoL inventory Short Form (SF)-36 to compare sino-nasal and HRQoL outcome in patients with or without allergies, previous nose surgeries, presence of pain, snoring, sleep apnea, usage of continuous positive airway pressure (cpap), and nose drop usage. *Results*: At the 3–6-month follow-up, patients with previous nasal surgery showed overall reduced subjective sino-nasal health, increased nasal and ear/head discomfort, increased visual impairment, and decreased psychological HRQoL (all *p* ≤ 0.026) after pituitary adenomectomy. Patients with pain before surgery showed a trend-level aggravated physical HRQoL (*p* = 0.084). *Conclusion*: Our data show that patients with previous nasal surgery have an increased risk of an aggravated sino-nasal and HRQoL outcome after pituitary adenomectomy. These patients should be thoroughly informed about potential consequences to induce realistic patient expectations. Moreover, the study shows that patients with moderately severe allergies, snoring, and sleep apnea (± cpap) usually do not have to expect a worsened sino-nasal health and HRQoL outcome.

## 1. Introduction

With an estimated prevalence between 10% and 17% in the general population, pituitary adenomas are among the more common intracranial tumors [1]. In recent years, transsphenoidal endoscopic pituitary adenoma resection has become the primary treatment option for almost all pituitary adenomas requiring surgery, due to its minimally invasive surgical approach [2,3]. The recent advances in the endoscopic approach have led to a significant reduction in the probability and extent of postoperative sequelae, especially in light of continuous refinements of surgical techniques and equipment, as well as the usage of rhino-septal splints [4,5]. Despite these advances, surgery through the transsphenoidal pathway is still associated with a deterioration of sino-nasal health [6]. This is an important fact to consider, since sino-nasal health has a pronounced impact on patients’ HRQoL [7]. To date, short-term, as well as persistent long-term effects of transsphenoidal endoscopic pituitary adenoma resection on sino-nasal health and HRQoL have been observed in patients with skull base lesions [8,9]. In view of the persistence of the long-term effects on sino-nasal health, the question arises whether persistent pre-surgery patient health variables (i.e., allergies, previous nose surgeries, presence of pain, snoring, sleep apnea with or without usage of cpap, and nose drop usage) might also affect sino-nasal health and HRQoL in the follow-up in this patient cohort. Since this matter is of utmost importance for patient education and future studies, we examined sino-nasal health (SNOT-NC) and HRQoL (SF-36) in the follow-up after bi-nostril transsphenoidal endoscopic adenoma resection and compared patients based on their prior medical history and health status.

## 2. Materials and Methods

### 2.1. Questionnaires

#### 2.1.1. Health-Related Quality of Life (HRQoL)

The German version of the self-report questionnaire SF-36 [10,11] was employed to assess HRQoL. The SF-36 is utilized as an outcome measure for the evaluation of health treatments. It measures HRQoL with 36 items in 8 z-standardized subdomains, i.e., general health, vitality, bodily pain, mental health, role physical, role emotional, as well as physical and social functioning, with higher scores indicating a greater HRQoL. Moreover, the SF-36 allows the reporting of summary measures for physical and mental health, based on the addition of the weighted scores of the 8 subdomains. In this study, we only examined the summary scores.

#### 2.1.2. Sino-Nasal Outcome Test for Neurosurgery (SNOT-NC)

The SNOT-NC [7] is a self-report questionnaire, developed for the assessment of the (subjective) clinical sino-nasal outcome of patients undergoing transsphenoidal endoscopic skull base surgery. The questionnaire is partially derived from the SNOT-22 questionnaire [12,13,14], but was extended with additional items and domains in order to include potential consequences of this particular neurosurgical approach. By means of 23 items, the domains Nasal Discomfort, Sleep Problems/Reduced Productivity, Ear and Head Discomfort, Visual Impairment, and a sino-nasal total score are examined.

#### 2.1.3. Clinical ad hoc Questions

To identify potential risk factors and pre-surgery impairments of nasal and overall health, which might impact the sino-nasal and HRQoL outcome measures, participants were asked to answer a clinical ad hoc questionnaire with 7 items. In this questionnaire, the presence of allergies, previous nose surgeries (nasal septum, paranasal sinuses, and turbinates), the presence of any pain, snoring, sleep apnea with or without usage of cpap, and nose drop usage (all yes or no) were queried.

### 2.2. Statistical Analyses

All statistical analyses were conducted with SPSS 27. The normal distribution of data was controlled for with the Kolmogorov–Smirnov test. In case of normal distribution, independent t-tests were used to compare groups based on the clinical ad hoc questions. The Leven’s test was used to control for equal variance. Non-normally distributed data were compared with Mann–Whitney U tests. Effect sizes were estimated using Cohen’s d (small < 0.2, medium < 0.5, and large effect > 0.5). For descriptive statistics, the mean and standard error of the mean are reported.

### 2.3. Sample

All 81 patients underwent surgery for the resection of pituitary adenoma via a bi-nostril transsphenoidal endoscopic approach and were consecutively treated at the Department of Neurosurgery and Spine Surgery at the University Hospital Essen between 1 August 2016 and 31 August 2021. Patients were only included in the 3–6-month follow-up if informed written consent was given. The study was conducted in accordance with the declaration of Helsinki and was approved by the institutional review board of the Medical Faculty of the University Hospital Essen (identification number: 14-5791-BO).

## 3. Results

### 3.1. Sample Characteristics

In total, 81 patients (40 female; mean age: 52.94 ± 1.64 years) participated in the study. A total of 4.94% of all patients classified for Cushing’s disease and 9.88% of all patients classified for acromegaly. Two patients were subsequently treated due to cerebrospinal fluid leakage. A total of 23.5% of patients had a KNOSP score of 0, 24.6% of 1, 14.9 of 2, 13.5 of 3 A or B, and the rest of the patients had a KNOSP score of 4.

### 3.2. SNOT-NC

**Figure 1:**
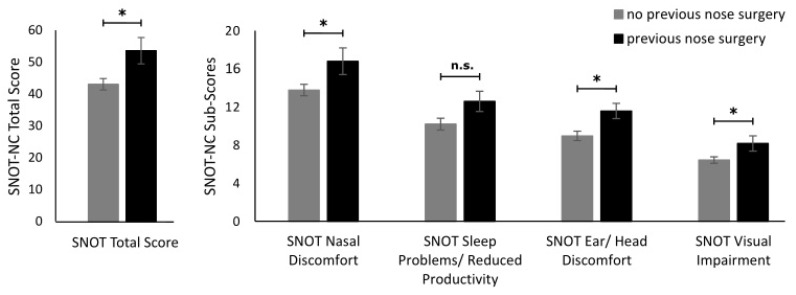
Illustration of the SNOT-NC Total Score (left side) and SNOT-NC Sub-Scores (right side) comparison for patients with (black) and without previous nasal surgeries (grey). Asterisks * mark significant differences.

SNOT-NC scores were analyzed in the follow-up to examine whether the subjective sino-nasal outcome is affected by pre-surgery patient health variables. Sino-nasal outcome scores were only affected in patients with previous nasal surgeries (*n* = 19). These patients exhibited significantly higher SNOT-NC total scores (53.57 ± 4.13 vs. 43.04 ± 1.79; t = 2.67; *p* = 0.009; d = 0.701), higher nasal discomfort (16.79 ± 1.40 vs. 13.77 ± 0.60; t = 2.27; *p* = 0.026; d = 0.595), higher ear/head discomfort (11.57 ± 1.13 vs. 8.96 ± 0.49; t = 2.12; *p* = 0.042; d = 0.632), and higher visual impairment (8.16 ± 0.80 vs. 6.42 ± 0.34; t = 2.32; *p* = 0.023; d = 0.608) than patients without previous surgeries. All other group comparisons based on pre-surgery variables yielded no significant group differences (all t ≤ 1.89; all *p* ≥ 0.063), even though trend-level significantly higher sleep problems/reduced productivity in patients with previous nasal surgeries (12.58 ± 1.06 vs. 10.19 ± 0.62; t = 1.89; *p* = 0.063; d = 0.248) were observed (see Figure 1). KNOSP scores did not differ between patients with or without tumors (without 1.88 ± 0.20 vs. with 1.82 ± 0.33; Z = −0.04; *p* = 0.969).

Moreover, trend-level higher visual impairment in patients with previous pain (6.49 ± 0.38 vs. 7.73 ± 0.62; t = 1.71; *p* = 0.092, d = −0.426) and trend-level lower visual impairment in patients with previous snoring (7.39 ± 0.54 vs. 6.25 ± 0.34; t = 1.78; *p* = 0.080; d = 0.394) were observed. All descriptive values are depicted in Table 1.

### 3.3. SF-36

SF-36 summary scores were analyzed in the follow-up based on the clinical ad-hoc questions for pre-surgery variables to examine whether HRQoL is affected. HRQoL summary scores were only affected in patients with previous nasal surgeries (*n* = 17), which exhibited a significantly decreased psychological HRQoL (37.31 ± 2.95 vs. 47.65 ± 1.44; t = 3.36; *p* = 0.001; d = 0.927) in comparison with patients without previous surgeries. All other group comparisons based on pre-surgery patient health variables yielded no significant group differences (all t ≤ 1.75; all *p* ≥ 0.084), even though trend-level significantly decreased physical HRQoL in patients reporting pain prior to the operation was observed (41.52 ± 2.97 vs. 46.48 ± 1.54; t = 1.75; *p* = 0.084; d = 0.475). All descriptive values are depicted in Table 2.

### 3.4. Correlation SNOT-NC and SF-36

**Figure 2:**
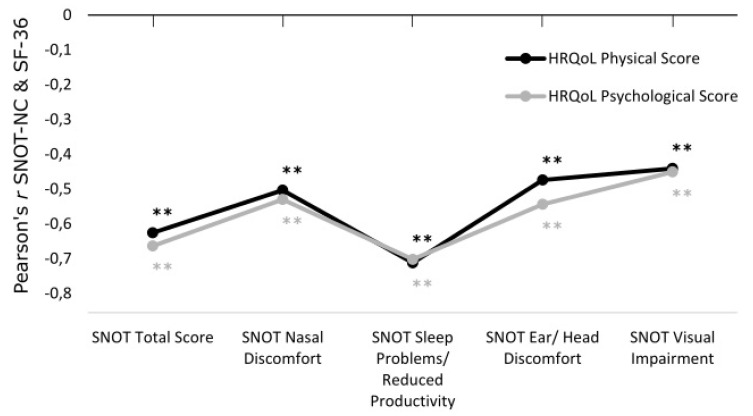
Pearsons’ *r* correlation coefficients for SNOT-NC Scores and SF-36 Psychological (black) and Physical HRQoL (grey). Double Asterisks ** mark highly significant correlations.

Correlations between the SNOT-NC and SF-36 were analyzed to examine the connection between sino-nasal health and HRQoL. Regarding the correlation between SNOT-NC and SF-36, medium to large correlations were observed between all scores of the SNOT-NC and the physical and psychological summary scores of the SF-36 (all r ≤ −0.441, all *p* < 0.001). This indicated a highly significant connection between decreased physical and psychological health and subjectively perceived sino-nasal health in the follow-up (see Figure 2).

## 4. Discussion

In this study, we focused on the question of whether pre-surgery patient health variables (i.e., patients’ prior medical history and health status) might additionally affect long-term sino-nasal health and HRQoL in patients after bi-nostril transsphenoidal endoscopic adenomectomy. The aim of this study was to identify specific patient health variables which should be examined to ensure an adequate patient education and to avoid a potential bias of data in future studies. To this end, we examined the impact of allergies, previous nose surgeries, presence of pain, snoring, sleep apnea with or without usage of cpap, and nose drop usage on subjective sino-nasal health and HRQoL in the follow-up after transphenoidal endoscopic adenoma resection.

The results show that previous nasal surgeries can have a major impact on sino-nasal outcome and postoperative HRQoL. Patients with previous nasal surgeries had an overall reduced subjective sino-nasal health, increased nasal and ear/head discomfort, increased visual impairment, as well as decreased psychological HRQoL. As aggravated sino-nasal health and HRQoL [9,15] as well as increased visual impairment [16] have also been shown in patients after revision surgery for adenoma resection, this underlines the importance of addressing the impact of previous nasal surgeries on sino-nasal health and HRQoL in patient education and future studies. These group differences were not driven by tumor size, as KNOSP scores did not differ between patients with and without previous surgeries. This underlines the importance of assessing previous nasal surgeries and thoroughly informing patients with previous nose surgeries about potential consequences of endoscopic pituitary surgery. Moreover, in line with previous literature [17,18], the data indicated on a trend-level basis that previous pain (regardless of pain location) might be related to deteriorated physical HRQoL and visual impairment in patients after pituitary adenoma resection. This leads to the advice to consider previous pain when examining HRQoL in pituitary adenoma patients, particularly in regards to the medium to large correlations between subjective sino-nasal health and HRQoL. Interestingly, the data showed no significant effect of allergies, snoring, sleep apnea with or without usage of cpap, and nose drop usage on subjective sino-nasal health and HRQoL. The lacking effect of allergies might be explained by the fact that HRQoL is usually affected by more severe types of allergies, e.g., respiratory allergies, food allergies, urticaria, and drug and sting allergies [19], while our patients only experienced mild to moderate symptoms. Similarly, out of 18 patients with sleep apnea, only 8 patients experienced sufficiently severe symptoms to require the usage of a cpap. As sino-nasal health has been shown to be correlated to the severity of sleep apnea [20] and effects of sleep apnea on HRQoL are highly correlated to day-time sleepiness [21], our results suggest that sleep apnea only plays a role in sino-nasal health and HRQoL in severely affected pituitary adenoma patients. Snoring might also not be a factor, as only mild subjective, but not objective, effects of clinical significant snoring on HRQoL are reported [22]. Similarly, pre-surgery nose drop usage might only indicate nasal problems, which are not severe enough to translate into post-operation sino-nasal health and HRQoL. As this study can, however, not rule out a potential impact of more severe symptoms, this issue should be addressed in a study examining sino-nasal health and HRQoL in more or less severely affected patients. A potential limitation is that pre-surgery health might be differently affected in patients with different types of pituitary adenomas. In acromegaly for example, an increased likelihood of sleep disorders and prominent changes in olfaction after surgery have been reported [23,24]. Due to the small sample size of patients that were diagnosed with acromegaly, we cannot entirely statistically rule out that the specific type of diagnosis might have biased our results. Hence, we strongly recommend that future studies should examine the role of pre-surgery health variables on sino-nasal outcome and quality of life in patients with different types of pituitary adenomas (e.g., cushing’s disease or acromegaly). Additionally, the growth direction of the tumor should be examined in future studies, as it might play a role in the treatment strategy and the outcome of the surgery [25].

## 5. Conclusions

To sum up, our study shows that previous nasal surgeries are an indicator of aggravated sino-nasal health and HRQoL in patients after transphenoidal endoscopic pituitary adenoma resection and should thus be addressed in patient education and in future studies. The data also suggest that pre-surgery pain relates to post-operation HRQoL and should thus be controlled for in future studies. Moreover, the study shows that patient groups who are, for the most part, moderately affected by allergies, snoring, or sleep apnea with or without usage of cpap usually do not have to expect a worsened sino-nasal health and HRQoL outcome. Future studies are recommended to examine how the severity of such health problems relates to subjective sino-nasal health and HRQoL after pituitary adenoma resection.

## Figures and Tables

**Table 1:** SNOT-NC Scores based on pre-surgery health variables.

Pre-Surgery Health Variable	Non-Existent (Mean ± SEM)	Existent (Mean ± SEM)	*t*-Value	*p*-Value	Cohen’s d
SNOT-NC Total Score					
Allergies	43.77 ± 1.84	46.35 ± 2.46	−0.88	0.379	−184
Previous nose surgeries	**43.04 ± 1.79**	**53.57 ± 4.13**	**−2.67**	**0.009**	**−0.701**
Presence of any pain	44.37 ± 1.94	48.56 ± 3.69	−1.08	0.285	−0.269
Snoring	46.47 ± 2.83	44.53 ± 2.00	0.560	0.577	0.124
Sleep apnea	45.36 ± 1.72	42.42 ± 2.57	0.95	0.350	0.201
Cpap	44.85 ± 1.64	43.75 ± 4.08	0.20	0.842	0.074
Nose drop usage	45.54 ± 2.47	44.49 ± 1.82	0.32	0.750	0.071
SNOT-NC Nasal Discomfort					
Allergies	13.89 ± 0.62	14.78 ± 0.82	−0.87	0.387	−0.181
Previous nose surgeries	**13.77 ± 0.60**	**16.79 ± 1.40**	**−2.27**	**0.026**	**−0.595**
Presence of any pain	14.21 ± 0.66	15.18 ± 1.20	−0.74	0.460	−0.186
Snoring	14.39 ± 0.95	14.56 ± 0.67	−0.15	0.882	−0.033
Sleep apnea	14.28 ± 0.59	14.03 ± 0.81	0.25	0.803	0.051
Cpap	14.22 ± 0.56	14.25 ± 0.90	−0.03	0.976	−0.006
Nose drop usage	14.79 ± 0.81	14.01 ± 0.62	0.71	0.482	0.158
SNOT-NC Sleep Problems/ Reduced Productivity					
Allergies	10.50 ± 0.56	11.65 ± 0.86	−1.16	0.249	−0.242
Previous nose surgeries	10.19 ± 0.62	12.58 ± 1.06	−1.89	0.063	−495
Presence of any pain	10.42 ± 0.63	11.64 ± 1.09	−0.99	0.324	−0.248
Snoring	10.73 ± 0.80	10.78 ± 0.74	−0.04	0.969	−0.009
Sleep apnea	11.13 ± 0.55	10.16 ± 0.89	0.80	0.426	0.204
Cpap	10.99 ± 0.52	10.38 ± 1.27	0.35	0.727	0.130
Nose drop usage	10.67 ± 0.78	11.04 ± 0.59	−0.34	0.732	−0.077
SNOT-NC Ear/ Head Discomfort					
Allergies	9.20 ± 0.52	9.52 ± 0.64	−0.39	0.701	−0.080
Previous nose surgeries	**8.96 ± 0.49**	**11.57 ± 1.13**	**−2.12**	**0.044**	**−632**
Presence of any pain	9.18 ± 0.50	10.62 ± 1.08	−1.21	0.235	−0.343
Snoring	10.32 ± 0.77	8.80 ± 0.52	1.63	0.107	0.361
Sleep apnea	9.52 ± 0.46	8.47 ± 0.73	1.04	0.303	0.264
Cpap	9.37 ± 0.44	9.25 ± 1.51	0.08	0.938	0.029
Nose drop usage	9.71 ± 0.80	9.16 ± 0.46	0.63	0.532	0.139
SNOT-NC Visual Impairment					
Allergies	6.48 ± 0.35	6.68 ± 0.49	−0.34	0.734	−0.071
Previous nose surgeries	**6.42 ± 0.34**	**8.16 ± 0.80**	**−2.32**	**0.023**	**−0.608**
Presence of any pain	*6.49 ± 0.38*	*7.73 ± 0.62*	*−1.71*	*0.092*	*−0.426*
Snoring	*7.39 ± 0.54*	*6.25 ± 0.34*	*1.78*	*0.080*	*0.394*
Sleep apnea	6.73 ± 0.33	5.79 ± 0.48	0.11	0.190	0.337
Cpap	6.60 ± 0.32	6.13 ± 0.99	0.44	0.658	0.164
Nose drop usage	6.25 ± 0.40	6.67 ± 0.36	−0.67	0.505	−0.150

Descriptive values for SNOT-NC group comparisons based on pre-surgery health variables for all SNOT-NC scores (SNOT-NC Total Score; SNOT-NC Nasal Discomfort; SNOT-NC Sleep Problems/ Reduced Productivity; SNOT-NC Ear/ Head Discomfort; SNOT-NC Visual Impairment). **Bold**: significant, *italics* trend level significant group differences.

**Table 2:** SF-36: HRQoL based on pre-surgery health variables.

Pre-Surgery Health Variable	Non-Existent (Mean ± SEM)	Existent (Mean ± SEM)	*t*-Value	*p*-Value	Cohen’s d
Psychological HRQoL					
Allergies	46.44 ± 1.73	43.35 ± 2.29	1.08	0.282	0.260
Previous nose surgeries	**47.65 ± 1.44**	**37.31 ± 2.95**	**3.36**	**0.001**	**0.927**
Presence of any pain	46.48 ± 1.54	41.52 ± 2.97	1.55	0.125	0.420
Snoring	46.78 ± 1.98	44.10 ± 1.49	1.10	0.277	0.232
Sleep apnea	45.32 ± 1.40	45.55 ± 2.54	−0.08	0.941	−0.020
Cpap	45.39 ± 1.35	43.41 ± 2.99	0.46	0.644	0.172
Nose drop usage	46.96 ± 2.18	44.65 ± 1.72	0.74	0.461	0.194
Physical HRQoL					
Allergies	44.59 ± 1.68	42.98 ± 2.22	0.58	0.561	0.140
Previous nose surgeries	45.01 ± 1.57	40.54 ± 2.31	1.42	0.160	0.393
Presence of any pain	45.29 *±* 1.53	39.91 *±* 2.59	1.75	0.084	0.475
Snoring	43.08 ± 1.78	44.60 ± 1.56	−0.65	0.520	−0.137
Sleep apnea	43.32 ± 1.40	46.10 ± 1.71	−0.95	0.343	−0.251
Cpap	43.60 ± 1.33	45.91 ± 2.49	−0.55	0.583	−0.205
Nose drop usage	45.76 ± 2.17	43.32 ± 1.64	0.81	0.422	0.211

Descriptive values for HRQoL group comparisons based on pre-surgery health variables for the SF-36 summary scores (Psychological HRQoL; Physical HRQoL). **Bold**: significant -, *italics* trend level significant group differences.

## Data Availability

Not applicable.

## Abbreviations

HRQoL	health-related quality of life
SNOT[-NC]	sino-nasal outcome test [for neurosurgery]
SF-36	HRQoL inventory Short Form

## References

[1] Ezzat S., Asa S.L., Couldwell W.T., Barr C.E., Dodge W.E., Vance M.L., McCutcheon I.E. (2004). The prevalence of pituitary adenomas: A systematic review. Cancer.

[2] Ammirati M., Wei L., Ciric I. (2013). Short-term outcome of endoscopic versus microscopic pituitary adenoma surgery: A systematic review and meta-analysis. J. Neurol. Neurosurg. Psychiatry.

[3] Molitch M.E. (2017). Diagnosis and Treatment of Pituitary Adenomas: A Review. JAMA.

[4] Schlüter A., Ahmadipour Y., Vogelsang T., Kreitschmann-Andermahr I., Kleist B., Weller P., Holtmann L., Mattheis S., Lang S., Bergmann C. (2016). Evaluation of the application of rhino-septal splints in endoscopic transsphenoidal skull base surgery. Eur. Arch. Otorhinolaryngol..

[5] Swearingen B. (2012). Update on Pituitary Surgery. J. Clin. Endocrinol. Metab..

[6] Pledger C.L., Elzoghby M.A., Oldfield E.H., Payne S.C., Jane J.A. (2016). Prospective comparison of sinonasal outcomes after microscopic sublabial or endoscopic endonasal transsphenoidal surgery for nonfunctioning pituitary adenomas. J. Neurosurg..

[7] Ahmadipour Y., Müller O., Kreitschmann-Andermahr I., Mattheis S., Sure U., Hütter B.-O. (2020). Development, reliability, validity and sensitivity of the Sino-Nasal Outcome Test for Neurosurgery (SNOT-NC). Eur. Arch. Oto-Rhino-Laryngol..

[8] McCoul E.D., Bedrosian J.C., Akselrod O., Anand V.K., Schwartz T.H. (2015). Preservation of multidimensional quality of life after endoscopic pituitary adenoma resection. J. Neurosurg..

[9] Little A.S., Kelly D., Milligan J., Griffiths C., Prevedello D.M., Carrau R.L., Rosseau G., Barkhoudarian G., Otto B.A., Jahnke H. (2015). Predictors of sinonasal quality of life and nasal morbidity after fully endoscopic transsphenoidal surgery. J. Neurosurg..

[10] Stieglitz R.D., Bullinger M., Kirchberger I. (1998). SF-36. Fragebogen zum Gesundheitszustand. Göttingen: Hogrefe. Preis DM 298. Z. Für Klin. Psychol. Und Psychother..

[11] Ware J.E., Sherbourne C.D. (1992). The MOS 36-item short-form health survey (SF-36). I. Conceptual framework and item selection. Med. Care.

[12] Hopkins C., Gillett S., Slack R., Lund V.J., Browne J.P. (2009). Psychometric validity of the 22-item Sinonasal Outcome Test. Clin. Otolaryngol..

[13] Baumann I., Blumenstock G., DeMaddalena H., Piccirillo J.F., Plinkert P.K. (2007). Quality of life in patients with chronic rhinosinusitis: Validation of the Sino-Nasal Outcome Test-20 German Adapted Version. Hno.

[14] Piccirillo J.F., Merritt M.G., Richards M.L. (2002). Psychometric and clinimetric validity of the 20-Item Sino-Nasal Outcome Test (SNOT-20). Otolaryngol. Head Neck Surg..

[15] McCoul E.D., Anand V.K., Bedrosian J.C., Schwartz T.H. (2012). Endoscopic skull base surgery and its impact on sinonasal-related quality of life. Int. Forum Allergy Rhinol..

[16] Hayhurst C., Taylor P.N., Lansdown A.J., Palaniappan N., Rees D.A., Davies J.S. (2020). Current perspectives on recurrent pituitary adenoma: The role and timing of surgery vs adjuvant treatment. Clin. Endocrinol..

[17] Breivik H., Borchgrevink P.C., Allen S.M., Rosseland L.A., Romundstad L., Hals E.K.B., Kvarstein G., Stubhaug A. (2008). Assessment of pain. Br. J. Anaesth..

[18] Finan P.H., Goodin B.R., Smith M.T. (2013). The Association of Sleep and Pain: An Update and a Path Forward. J. Pain.

[19] Baiardini I., Braido F., Brandi S., Canonica G.W. (2006). Allergic diseases and their impact on quality of life. Ann. Allergy Asthma Immunol..

[20] Kuan E.C., Tajudeen B.A., Peng K.A., Wang M.B. (2015). Sinonasal outcomes in obstructive sleep apnea syndrome. Laryngoscope.

[21] Silva G.E., Goodwin J.L., Vana K.D., Quan S.F. (2016). Obstructive sleep apnea and quality of life: Comparison of the SAQLI, FOSQ, and SF-36 questionnaires. Southwest J. Pulm. Crit. Care.

[22] Stoohs R.A., Knaack L., Blum H.-C., Janicki J., Hohenhorst W. (2008). Differences in clinical features of upper airway resistance syndrome, primary snoring, and obstructive sleep apnea/hypopnea syndrome. Sleep Med..

[23] Pertichetti M., Serioli S., Belotti F., Mattavelli D., Schreiber A., Cappelli C., Padovani A., Gasparotti R., Nicolai P., Fontanella M.M. (2020). Pituitary adenomas and neuropsychological status: A systematic literature review. Neurosurg. Rev..

[24] Netuka D., Masopust V., Fundová P., Astl J., Školoudík D., Májovský M., Beneš V. (2019). Olfactory Results of Endoscopic Endonasal Surgery for Pituitary Adenoma: A Prospective Study of 143 Patients. World Neurosurg..

[25] Micko A., Agam M.S., Brunswick A., Strickland B.A., Rutkowski M.J., Carmichael J.D., Shiroishi M.S., Zada G., Knosp E., Wolfsberger S. (2022). Treatment strategies for giant pituitary adenomas in the era of endoscopic transsphenoidal surgery: A multicenter series. J. Neurosurg..

